# Outcomes of Staged Treatment for Complex Distal Radius Fractures

**DOI:** 10.7759/cureus.3273

**Published:** 2018-09-10

**Authors:** Brooks Ficke, Erin F Ransom, Matthew C Hess, Andrew S Moon, Haley M McKissack, Ashish Shah, Nileshkumar Chaudhari

**Affiliations:** 1 Orthopaedic Surgery, Resurgens Orthopaedics, Atlanta, USA; 2 Orthopaedic Surgery, University of Alabama at Birmingham, Birmingham, USA; 3 Orthopaedic Surgery, Tufts Medical Center, Tufts University School of Medicine, Boston, USA

**Keywords:** distal radius fracture, staged treatment, external fixation, delayed fixation, damage control

## Abstract

Introduction

Distal radius fractures are common, but the results and complications of treatment with early external fixation and staged open reduction internal fixation have not been previously reported.

Materials and methods

Patients who received staged distal radius fracture treatment from 1/1/2008 to 12/31/2015 at the University of Alabama at Birmingham were identified. Patient, injury, and treatment characteristics, as well as complications, were collected from the medical record.

Results

There were 50 fractures in 47 patients, with mean follow-up of 9.3 months. Thirty-eight were open and 45 were intra-articular. For definitive treatment, 41 received a volar approach and nine a dorsal approach. Twenty wrists experienced one or more complications, including two non-unions. Five patients developed infections – one Kirschner wire site infection, one external fixator (ex-fix) pin site infection, and three deep infections. All deep infections occurred in tobacco users. The rate of deep infection with volar approach was 2.4%, compared to 22.2% with dorsal approach. Ex-fix pin sites overlapped radiographically with the plate in 20 fractures, with three deep infections in this group (15%) and no deep infections in the group without overlap. None of these differences reached statistical significance.

Conclusions

This protocol results in reliable healing of complex fractures, with a 96% union rate. However, 40% sustained complications. We conclude that this protocol is useful for temporizing complex fractures but caution that the complication rate is high. Since recent literature indicates that low-grade open distal radius fractures do not require emergent debridement and that immediate internal fixation is safe, complications might be avoided by restricting this protocol to complex or physiologically unstable patients.

## Introduction

Distal radius fractures are among the most common fractures treated by hand and upper extremity surgeons. Treatment options include splinting, external fixation, and open reduction internal fixation through various approaches [1]. The care of polytrauma patients with distal radius fractures often involves multiple surgeons, particularly when open fractures, irreducible deformity, or acute carpal tunnel syndrome dictate immediate intervention. Prioritization of other injuries, patient condition, wound contamination, soft tissue status, fracture complexity, operative timing, and specialist surgeon availability may preclude initial definitive treatment, leading the surgeon to choose damage control type procedures. In this setting, splinting or external fixation may be used as temporizing measures to maintain length and alignment until definitive open reduction internal fixation can be performed. External fixation may be indicated when soft tissues require frequent monitoring or care, when adequate reduction cannot be maintained, after a carpal tunnel release or neurovascular repair to prevent instability and neurovascular injury, when patient instability precludes a long procedure for open reduction internal fixation, or when it would simplify the care of a polytrauma patient.

The staged treatment of distal radius fractures with early external fixation followed by delayed open reduction internal fixation has not been extensively studied, and indications for its use are not well defined. Glueck et al. used this protocol in six of their 42 patients with open distal radius fractures and reported no infections [2]. Kurylo et al. used this protocol in five of their 32 patients with open distal radius fractures and noted no infections but an increased rate of other complications requiring secondary surgical procedures [3].

This retrospective study details our experience treating distal radius fractures with a staged protocol of external fixation followed by open reduction internal fixation (ORIF). We hypothesized that the use of a dorsal approach for definitive fixation, radiographic overlap of the external fixator pin sites with the final plate location, length of time spent in the external fixator, smoking, illegal drug use, and diabetes would be associated with infection.

## Materials and methods

Institutional Review Board approval and waiver of informed consent requirement was obtained at the University of Alabama in Birmingham before commencing this study. All patients receiving staged treatment of distal radius fractures with external fixation followed by open reduction internal fixation from 1/1/2008 through 12/31/2015 were identified using a Current Procedural Terminology (CPT) (American Medical Association, Chicago, Illinois) code search. Screening with CPT codes 25605, 25606, 25607, 25608, and 25609 identified 1,218 operatively treated distal radius fractures within the indicated dates. By searching this data set for patients also coded with CPT codes 20694, 25620, 11010, 11011, and 11012, 96 potential patients were initially identified. Patients were included if they had a distal radius fracture that was treated initially with external fixation and later converted to open reduction internal fixation. Patients were excluded if they did not have a distal radius fracture (18), were not treated with the staged protocol (14), had less than two months of follow-up (14), were placed into an external fixator for existing infection (two), or were under age 18 (one). Patients with less than three months follow-up were excluded from the analysis of non-union rate. Forty-seven patients and 50 wrists treated were included in the study.

**Figure 1:**
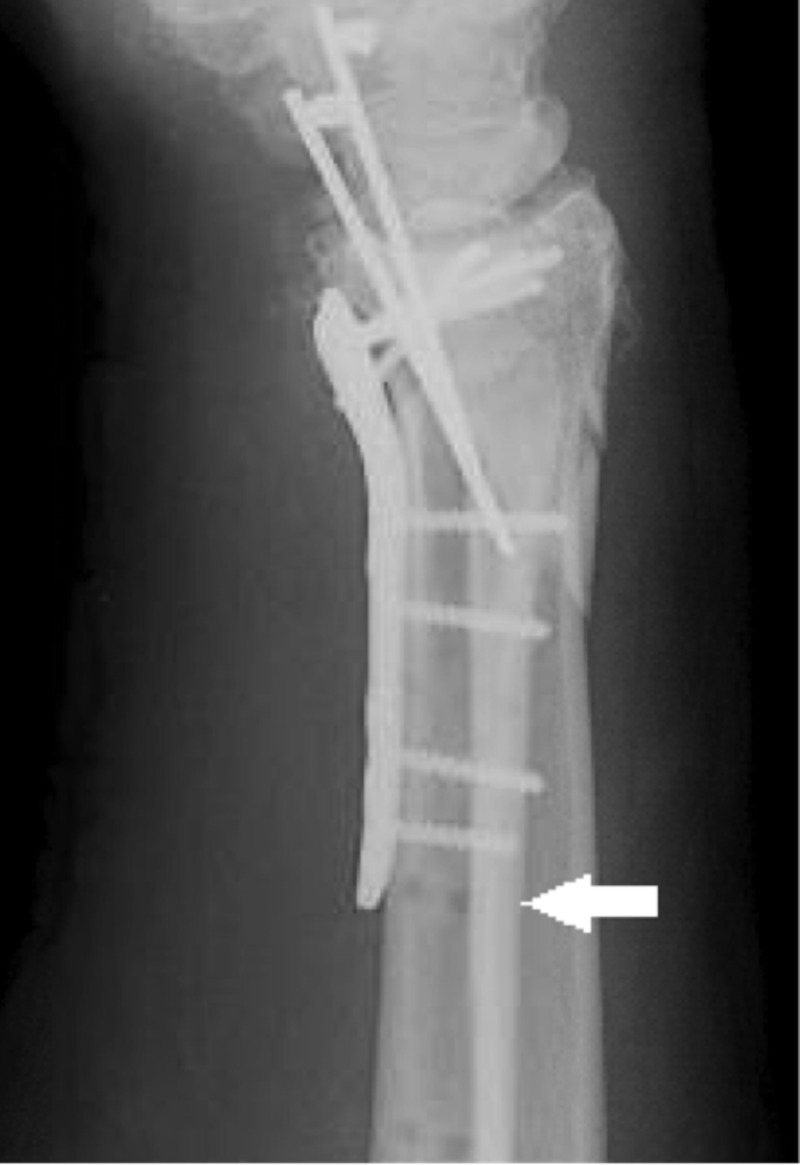
Lateral wrist X-ray demonstrating overlap of the volar plate with the track from the former pin site (white arrow).

Patient characteristics, injury characteristics, and complications were collected from the electronic medical record. Patient characteristics included: sex, polytrauma status, diagnosis of diabetes, any source of immunosuppression, and history of smoking or drug use. Injury characteristics included: mechanism, Arbeitsgemeinschaft für Osteosynthesefragen/American Orthopaedic Trauma Association (AO/OTA) classification, Gustilo classification, presence of contamination of open injuries as reported by the treating surgeon, and presence of bone loss (through open wounds or dorsal comminution) upon retrospective review of operative reports and imaging. Treatment characteristics included: surgical approach used on conversion to open reduction internal fixation and whether the external fixation pin sites overlapped with the final plate location on post-operative X-rays (Figure 1). Complications included: unplanned subsequent procedures, delayed union (not healed after three months) or nonunion (not healed after six months), wrist or finger stiffness as subjectively noted by the treating physician during post-operative visits, nerve injuries, hardware complications, tendon rupture, infection, and new pathologic conditions in the hand. Fisher's Exact Test was used to analyze categorical data. The percentage of wrists that sustained a deep tissue infection was calculated for each of five categorical variables. P-values were determined to compare whether a significant association existed between presence of deep tissue infection and five categorical variables: dorsal versus volar surgical approach, presence of pin site overlap, previous diagnosis of diabetes, tobacco use, and drug use. A p-value of less than 0.05 was considered statistically significant. Wilcoxon two-sample test was used to evaluate infection and length of time spent in the external fixator.

## Results

There were 33 male and 14 female patients with a mean follow-up of 9.3 months (range: 2–52) (Table 1). Mechanism of injury, fracture characteristics, and treatment characteristics are detailed in Table 2. Indications for use of the protocol were varied, and included open fractures, traumatized soft tissues, acute carpal tunnel syndrome, radial artery repair, and inability to maintain reduction with a splint.

**Table 1:** Patient demographics.

Characteristic	Number of Patients
Male	33
Female	14
Diabetes	6
Tobacco Use	21
Drug Use	4
Immunocompromised	1
Polytrauma	32

**Table 2:** Injury and fixation characteristics. AO/OTA: Arbeitsgemeinschaft für Osteosynthesefragen/American Orthopaedic Trauma Association

Fracture Specifics	Number of Wrists
Etiology	
Motorcycle Collision	17
Fall from Height	12
Motor Vehicle Collision	11
Fall from Standing	4
Bicycle	3
Gunshot	2
Crush	1
AO/OTA Classification	
A	5
B	4
C	41
Closed Fracture	12
Gustilo Classification	
1	15
2	17
3	6
Contamination	6
Bone Loss	27
Surgical Approach	
Volar	41
Dorsal	9
Pin Site Overlap	20

Patients spent a mean of 9.2 days in the external fixator (range: 1–59 days). Mean time among those who developed deep tissue infections was four days, and mean among those without infection was 9.5 days (p = 0.22). Complication rates are shown in Table 3. Among 46 fractures with three months or longer follow-up, there were two non-unions. These occurred in the setting of a closed AO 23-B2 fracture that displaced after hardware removal at six months post-operatively from initial fixation and a grade 1 open AO 23-C3 fracture that developed a deep tissue infection which was treated with antibiotic spacer and has not undergone further procedures due to cardiac comorbidities. The single nerve injury was to the dorsal sensory branch of the radial nerve. Twenty-five (50%) wrists had postoperative stiffness noted during follow-up, which was not included in the analysis of complications due to its subjective nature and the expectation of some degree of stiffness after severe trauma.

**Table 3:** Complications by category.

Complication	Number of Wrists
Delayed Union	3
Non-union	2
Infection	
Deep Tissue	3
Kirschner Wire	1
Pin Site	1
Hardware Complication	
Painful Hardware	5
Sigmoid Notch Erosion	1
Loss of Reduction	1
2^nd^ Metacarpal Fracture	1
Unplanned Procedures	
Infection-Related	3
Hardware Removal	4
Delayed Closure	2
Carpal Tunnel Release	2
Tenolysis	2
Hematoma Debridement	1
Neurolysis	1
Tendon Transfer	1
Hardware Revision	1
Ulnar Shortening Osteotomy	1
Pathologic Conditions	
Stenosing Tenosynovitis	2
Heterotopic Ossification	1
Dupuytren’s Cords	1
Tendon Rupture	2
Nerve Injury	1

Five (10%) wrists had an infection – one had a Kirschner wire site infection, one had an ex-fix pin site infection prior to conversion, and three had deep tissue infections. All were in open fractures. The infected Kirschner wire was removed at bedside, and the infection resolved uneventfully after a course of oral antibiotics. The pin site infection was treated with oral antibiotics and removal of the external fixator in clinic followed by bridge plating eight days later, and the patient had no signs of infection at 10-month follow-up. In all three deep tissue infections cultures revealed methicillin-resistant Staphylococcus aureus.

Associations between categorical variables and deep tissue infection are shown in Table 4. All three deep tissue infections occurred in wrists with pin site overlap and tobacco use. Of all wrists with pin site overlap, 15% sustained deep tissue infection. No deep tissue infections occurred in wrists without pin site overlap. Although significance was approached, association between the presence of pin site overlap and deep tissue infection was not statistically significant (p = 0.06). Fourteen percent of wrists of tobacco users sustained deep tissue infection, while zero non-tobacco users sustained deep tissue infection. Significance was approached, but no statistically significant association was found between deep tissue infection and tobacco use (p = 0.08). Among wrists fixed with the dorsal approach, 22.2% sustained deep tissue infection, compared to 2.4% of wrists fixed with the volar approach. Again, significance was approached but not reached for association between deep tissue infection and surgical approach (p = 0.08). No statistically significant association was found between deep tissue infection and patient drug use (p = 0.23) or deep tissue infection and concurrent diabetes (p = 0.41).

**Table 4:** Analysis of variables associated with deep tissue infections.

Variable	Number of Wrists with Deep Tissue Infections	Percent of Wrists with Deep Tissue Infections (%)	p-value
Approach			
Dorsal	2	22.2	p = 0.08
Volar	1	2.4	
Pin Site Overlap			
Yes	3	15	p = 0.06
No	0	0	
Diabetes			
Yes	1	12.5	p = 0.41
No	2	4.8	
Tobacco Use			
Yes	3	14	p = 0.08
No	0	0	
Drug Use			
Yes	1	25	p = 0.23
No	2	4.3	

The first deep tissue infection presented two months post-operatively in a 30-year-old smoker with a contaminated grade 3A open injury after a motorcycle crash treated with a dorsal bridge plate one day after external fixation. His infection was treated with hardware removal, antibiotic bead placement, and intravenous antibiotics, with later bead removal. He had no signs of residual infection at 19-month follow-up.

The second deep tissue infection presented four months post-operatively in a 71-year-old diabetic smoker with a grade 1 open injury after a fall treated with a volar approach and styloid percutaneous pinning four days after external fixation. The percutaneous pins had no signs of infection on removal at two weeks. His infection was treated with hardware removal, antibiotic spacer placement, and intravenous antibiotics. He was minimally symptomatic at 13-month follow-up despite a non-union and had severe cardiac co-morbidities that precluded further surgery.

The third deep tissue infection presented four months post-operatively in a 28-year-old smoker and drug abuser who sustained a grade 2 open injury due to a gunshot wound treated with a dorsal bridge plate seven days after external fixation. His fracture healed but he complained of dorsal hand pain and swelling, and X-rays revealed loosening of multiple screws. Gross purulence was found during hardware removal. He was treated with antibiotic beads and intravenous antibiotics. After bead removal and extensor pollicis longus tenolysis he had no signs of residual infection.

## Discussion

Distal radius fractures, though common, can be complex injuries. We reviewed 50 cases of wrists treated with a staged protocol of external fixation followed by open reduction internal fixation. We found that staged treatment of distal radius fractures is an effective way of treating complex fractures, with a 96% union rate. However, there is a significant risk of complications, with 40% of patients suffering at least one complication, which highlights the dangers associated with these complex fractures. It should be noted that three of the complications (a pin site infection, a second metacarpal fracture, and an injury to the superficial branch of the radial nerve) could be solely attributed to the use of an external fixator. In addition to the high rate of complications, half of the patients had some degree of post-operative stiffness, which might be expected given the high-energy nature of many of the injuries in this study but should be aggressively addressed during the early post-operative period. These findings are consistent with the experience of Kurylo, who noted high rates of secondary procedures related to stiffness and soft tissue scarring in patients treated with a staged protocol [3].

The rate of pin site infection is difficult to compare with prior studies, which note up to a 30% incidence of pin tract infection, because the fixators were generally removed early in the treatment course (mean: 9.2 days) [1]. Patients with deep tissue infections were in their external fixators for one, four, and nine days, and none had clinical signs of pin site infection during their treatment course.

The overall deep tissue infection rate among open fractures (7.9%) in this study is comparable with that found in other studies of open fractures. Kurylo et al. found no infections in 32 patients, Kaufman et al. had a 5% rate of infection in 21 patients, Glueck et al. had a 7% rate of infection in 42 patients, and Rozental et al. reported a 44% rate of infection in 18 patients [2-5]. Glueck et al. also noted that level of contamination was associated with infection [2]. In this study, there was no association between level of contamination or Gustilo classification with deep infection. Though statistical significance was not reached, there were trends towards association with deep infection for both the dorsal surgical approach and pin site overlap. There may be a risk of contamination when the external fixator pin sites are included in the incision for the dorsal approach. With a volar approach, the contaminated tracts where the external fixator pins emerge from the skin can be isolated from the surgical wound. However, the presence of the bony pin tracts within the surgical site provides the possibility of bacterial contamination in the surgical wound, which is supported by the finding that no deep tissue infections occurred in the group without pin site overlap with the plate, regardless of approach. Additionally, the lack of any signs of pin site infection in the patients who developed deep infections is consistent with Mahan’s report that 74.8% of screw tips had positive cultures after removal, suggesting that sub-clinical pin site infection can contribute to deep infection in the setting of implanted hardware [6]. Based on these findings, we recommend careful planning during external fixator placement to avoid future pin site overlap, though sometimes overlap is unavoidable, particularly with fractures extending into the radial diaphysis.

This study has some limitations. Specifically, there were no pre-defined criteria for temporizing with external fixation, for conversion to internal fixation, or for operative treatment of complications. There was no control group with which to make comparisons, such as a group of patients treated with initial debridement and splinting followed by delayed open reduction internal fixation. Though the average follow-up was 9.3 months, we included patients with follow-up as short as two months to fully present the number and variety of complications experienced. Short follow-up is common in a trauma population. However, given that two of the deep tissue infections presented at four-to-five months post-operatively, it is possible that some infections might not have presented during the follow-up period. Several other types of complications typically present late, and so the analysis of the complication rate may be artificially low. Additionally, despite the high rate of complications, each type of complication was individually uncommon, and the total number of patients was small, limiting the ability to perform statistical analysis. Lastly, the cohort had a high percentage of complex cases, with the majority being open, intra-articular, or both, and therefore does not represent a typical group of patients with distal radius fractures. However, this staged protocol would not be required for most simple fractures, as evidenced by its use in only 5% of operatively treated distal radius fractures at our institution during the study dates.

Staged protocols for treatment of lower extremity injuries are well established, but there is less literature regarding staged treatment strategies for upper extremity injuries. It has been suggested that grade 1 or 2 open distal radius fractures do not require emergent irrigation and debridement, as several studies have shown no correlation between timing of surgery and development of infection [2,3,7]. Additionally, Kaufman et al. suggest that immediate open reduction internal fixation of open distal radius fractures is safe, even in geriatric patients [4]. Given this existing literature and our findings of high complication rates associated with the staged protocol, it may be appropriate to limit its use to situations where early definitive fixation is not feasible and where splinting is not felt to be both sufficient and safe as a temporizing measure following operative debridement (if indicated), reduction, and splinting. In this manner, those with well-reduced fractures and closed or grade 1 open injuries might avoid additional risks related to external fixation.

## Conclusions

This study reports the results of treatment of distal radius fractures with a staged protocol of initial external fixation followed by open reduction internal fixation. The staged protocol is effective at achieving union in complex fractures, but is associated with a high complication rate. We conclude that this protocol is a viable option for temporizing complex distal radius fractures but caution that it is associated with a high rate of complications.
